# Effects of *Fhb1*, *Fhb2* and *Fhb5* on *Fusarium* Head Blight Resistance and the Development of Promising Lines in Winter Wheat

**DOI:** 10.3390/ijms232315047

**Published:** 2022-11-30

**Authors:** Xuran Dai, Yiwen Huang, Xinhui Xue, Shuo Yu, Teng Li, Hongwei Liu, Li Yang, Yang Zhou, Hongjie Li, Hongjun Zhang

**Affiliations:** The National Engineering Laboratory for Crop Molecular Breeding, Institute of Crop Sciences, Chinese Academy of Agricultural Sciences, Beijing 100081, China

**Keywords:** *Triticum aestivum*, *Fusarium* head blight, pyramiding effect, double haploid, agronomic traits

## Abstract

The development of *Fusarium* head blight (FHB)-resistant winter wheat cultivars using the gene *Fhb1* has been conducted in northern China. Sumai 3, a Chinese FHB-resistant spring wheat cultivar, carries three FHB resistance genes: *Fhb1*, *Fhb2* and *Fhb5*. To better use these genes for increasing FHB resistance in northern China, it is necessary to elucidate the pyramiding effects of *Fhb1*, *Fhb2* and *Fhb5* in winter wheat backgrounds. Eight gene combinations involving *Fhb1*, *Fhb2* and *Fhb5* were identified in a double haploid (DH) population, and the effects on FHB resistance were evaluated in six tests. At the single gene level, *Fhb1* was more efficient than the other two genes in single-floret inoculation tests, whereas *Fhb5* showed better resistance than *Fhb1* and *Fhb2* under a natural infection test. Pyramiding *Fhb1*, *Fhb2* and *Fhb5* showed better FHB resistance than the other gene combinations. Forty-nine DH lines showing consistently better resistance than the moderately susceptible control Huaimai 20 in multiple tests were evaluated for main agronomic traits, and no difference in grain yield was found between the mean values of DH lines and the recipient parents Lunxuan 136 and Lunxuan 6, which are higher than those of recipient parent Zhoumai 16 and the donor parent Sumai 3 (*p* < 0.05). Based on the phenotypic and genomic composition analyses, five promising DH lines fully combined the FHB resistance of donor Sumai 3 and the elite agronomic traits from the recipient parents. This study elucidates the pyramiding effects of three FHB resistance genes and that the promising DH lines with resistance to FHB can be directly applied in wheat production or as parents in winter wheat breeding programs.

## 1. Introduction

*Fusarium* head blight (FHB) after leaf rust is the second-largest destructive disease in wheat (*Triticum aestivum* L.) production in the world, especially in Asia, North and South Americas and Europe [1,2,3]. This fungal disease has not only caused significant reductions in yield but has also deteriorated the quality by producing mycotoxins, especially deoxynivalenol (DON), which severely threatens human and livestock health [4,5]. In China, the annual wheat acreage infected by FHB is more than 4.5 million hectares, causing an average loss of over 3.41 million tons during 2000–2018 [6]. Due to changes in crop management practices and climates, FHB has spread from the southern to the northern wheat-producing regions of China. Almost all of the current wheat cultivars planted in northern China are highly susceptible to FHB, making the wheat production fully exposed to this devastating disease. Hence, the improvement of resistance to FHB becomes one of the most important breeding objectives in those wheat areas.

It is time-consuming and labor-intensive to improve FHB resistance only by conventional phenotypic selection due to its quantitative nature of inheritance and influence by environments [7]. Molecular marker-assisted selection (MAS) provides a feasible way to enhance breeding efficiency. According to host responses to pathogen infection, two types of resistance to FHB have been observed, i.e., resistance to either initial infection (Type I) or fungal spread within the spikes (Type II) [8]. To date, more than 432 quantitative trait loci (QTL) conferring FHB resistances were identified on all wheat chromosomes [3]. Seven of them are major genes and have been officially designated as *Fhb1*–*Fhb7*. The genes *Fhb1*, *Fhb2* and *Fhb5* from Sumai 3 and Wangshuibai and *Fhb4* from Wangshuibai were mapped on chromosomes 3BS, 4BL, 6BL and 5AS, respectively [3,8]. The other three genes are identified in the wild relatives of wheat, e.g., *Fhb3* from *Leymus racemosus* [9], *Fhb6* from *Elymus tsukushiensis* [10] and *Fhb7* from *Thinopyrum ponticum* [11], and have been transferred onto the wheat chromosomes 7AS, 1AS and 7DL, respectively [12]. Among those resistance genes, *Fhb1* and *Fhb7* have been cloned. The candidate genes of *Fhb1* were controversially described to a pore-forming toxin-like (*PFT*) gene [13] and a putative histidine-rich calcium-binding protein (*TaHRC*) [7,14]. In addition, *WFhb1-1*, encoding a putative membrane protein of 127 amino acids, was considered another candidate gene of *Fhb1* [15]. *Fhb7* encodes a glutathione S-transferase (GST) that limits the growth of the pathogen on spikes [16]. 

To discover genes underlying the FHB response network and elucidate the genetic mechanism, multiple omics-based approaches were widely used to investigate the differentially expressed genes, proteins and metabolites between resistant and susceptible cultivars after inoculation with *F*. *graminearum*. These differentially expressed products are mainly associated with defense-related cellular and molecular events, basal defense response, phytohormone-related defense signaling, antimicrobial substances and cell wall thickening [3]. The cell wall is the primary barrier preventing the invasion of fungal pathogens. Wall-associated kinases play an important role in the connection and communication between plant cell walls and cytoplasms [17]. Previous studies have reported that the cell wall structure-related genes *WheatPme-1* and *Glu-1* and the wall-associated receptor-like kinase *WAK2* gene can effectively increase resistance to FHB [18,19]. 

Resistance genes have been widely used to improve FHB resistance in Canada, Australia, America, Europe, and Asia [20]. In most cases, a single *Fhb1* gene could significantly improve FHB resistance, but its effect depended on genetic backgrounds [12,21,22]. Pyramiding resistance genes were more efficient and stable in increasing resistance than the use of a single gene [8,23]. In a susceptible PH691 genetic background, the gene combinations of *Fhb4* + *5* and *Fhb1 + 2* signficanly increased Types I and II resistance to FHB, respectively [8]. Similarly, backcrossing progenies stacking *Fhb1*, *Fhb4* and *Fhb5* from Wangshuibai reduced disease severity by 95% of their recurrent parents [24]. 

The fast movement of FHB northward in China has attracted wide attention for wheat breeders. The most important limitation of such a breeding program is the shortage of adapted resistant sources and effective methods of selection and disease assessments. Currently, the available resistant sources of FHB are mainly spring wheat cultivars from the Middle and Low Yangtze Winter Wheat Zone (MLWZ) of China. Due to the difference in ecological type, these resistant sources are not readily used in winter wheat production in northern China. Sumai 3 is a spring wheat cultivar that is highly resistant to FHB, conferred by the genes *Fhb1*, *Fhb2* and *Fhb5*. We initiated a project to improve FHB resistance by incorporating *Fhb1* into the locally adapted winter wheat cultivars. The FHB resistance of *Fhb1*-carrying progenies was significantly improved in comparison with their recurrent parents [22,25,26]. The objectives of the present study were to (1) compare the effectiveness of single gene and pyramiding *Fhb1*, *Fhb2* and *Fhb5* in winter wheat double haploid (DH) lines; (2) evaluate the main agronomic traits of selected FHB-resistant DH lines; and (3) analyze the genomic composition of the five promising DH lines. 

## 2. Results

### 2.1. Identification of Fhb1, Fhb2 and Fhb5 in Parents and DH Lines

**Figure 1:**
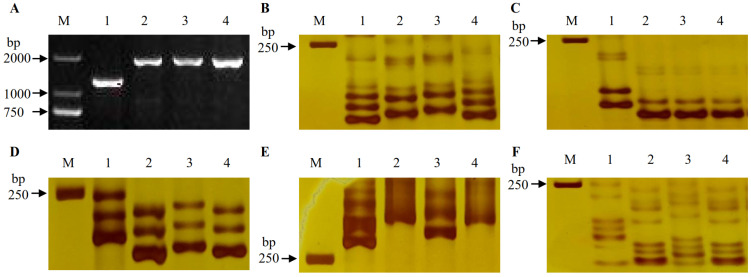
Polymerase chain reaction (PCR) amplification of *Fhb1* by *TaHRC-GSM* (**A**), *Fhb2* by *Xwmc397* (**B**) *Xwmc398* (**C**), *Fhb5* by *Xgwm304* (**D**), *Xhbg394* (**E**) and *Xwmc705* (**F**). M: the deoxyribonucleic acid (DNA) size standard (in bp). Lanes 1, 2, 3 and 4: Sumai 3, Zhoumai 16, Lunxuan 136 and Lunxuan 6, respectively.

A 1.4 kb fragment was amplified from Sumai 3, demonstrating that it carries the *Fhb1* resistance allele, while the *Fhb1* susceptibility allele indicated by a 2.0 kb fragment was detected in the three recipient parents (Figure 1A). The *Fhb2* resistance allele, defined by the amplification of the 155 and 160 bp target bands using the markers *Xwmc397* (Figure 1B) and *Xwmc398* (Figure 1C), respectively, was detected only in Sumai 3. The 220, 270 and 160 bp target bands specific for the *Fhb5* resistance allele were amplified in Sumai 3 using the markers *Xgwm304* (Figure 1D), *Xhbg394* (Figure 1E) and *Xwmc705* (Figure 1F), respectively. Hence, these markers were used to identify the expected alleles from the DH lines. Eight gene combinations of *Fhb1*, *Fhb2* and *Fhb5* (Supplementary Table S1) were selected to evaluate the FHB resistance and compare the pyramiding effects in six tests. 

### 2.2. Effects of a Single Gene and Gene Combinations of Fhb1, Fhb2 and Fhb5 on the FHB Resistance

**Figure 2:**
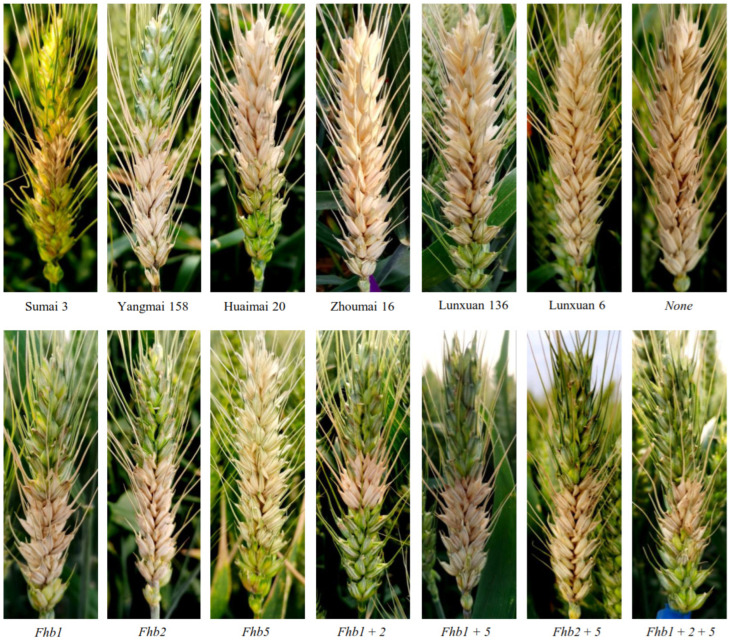
Performance of *Fusarium* head blight resistance in the double haploid (DH) lines pyramiding *Fhb1*, *Fhb2* and *Fhb5*, evaluated by the single-floret inoculation at 2021HN.

**Figure 3:**
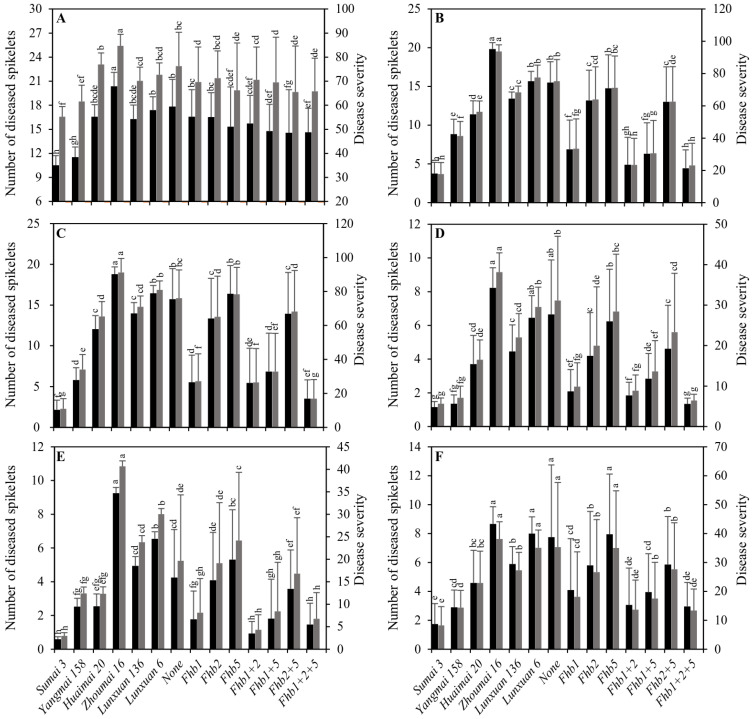
Comparison of the number of diseased spikelets (black columns) and the disease severity (grey columns) in the double haploid (DH) lines pyramiding *Fhb1*, *Fhb2* and *Fhb5* at 2020FJ (**A**), 2021HN (**B**), 2021BJ (**C**), 2021GH (**D**), 2022HN (**E**) and 2022GH (**F**). Multiple comparisons were performed using the least significant difference (LSD) test, and different letters above the standard deviation bars indicate significant difference among genotypes in a single test at *p* < 0.05.

Analysis of variance (ANOVA) showed that the mean squares of genotypes, tests and their interaction were significant for a number of diseased spikelets and disease severity (Supplementary Table S2). The number of diseased spikelets or the disease severity for DH lines were significantly correlated in different tests (Supplementary Table S3). The difference in the FHB resistance was observed among the four controls (*p* < 0.05) (Figure 2 and Figure 3). The highly and moderately resistant controls Sumai 3 and Yangmai 158 showed better FHB resistance, which was manifested by fewer diseased spikelets and lower disease severity scores than the moderately and highly susceptible controls Huaimai 20 and Zhoumai 16 in six tests (Figure 3). Meanwhile, Huaimai 20 had fewer diseased spikelets and a lower disease severity than Zhoumai 16 (*p* < 0.05) in all tests. The difference in FHB resistance among the four controls demonstrated that the single-floret inoculation and the natural infection were successful in all tests and that the disease pressures were suitable for disease assessments. 

The DH lines carrying *Fhb1*, *Fhb2*, *Fhb1 + 2*, *Fhb1 + 5*, *Fhb2 + 5* and *Fhb1 + 2 + 5* showed fewer diseased spikelets and lower disease severity scores than the recipient parents Zhoumai 16 (highly susceptible control) and Lunxuan 6 and the DH lines carrying the susceptibility alleles in the three loci in six tests (Figure 3). In comparison with another recipient parent Lunxuan 136, the DH lines with *Fhb5*, *Fhb1 + 2*, *Fhb1 + 5*, *Fhb2 + 5* and *Fhb1 + 2 + 5* exhibited a lower number of diseased spikelets and a lower disease severity at 2020FJ (Figure 3A), and those with *Fhb1*, *Fhb1 + 2*, *Fhb1 + 5* and *Fhb1 + 2 + 5* showed better FHB resistance under the single-floret inoculation tests (Figure 3B–F). 

Under the natural infection test in the trial of 2020FJ, the DH lines carrying *Fhb5* had a lower number of diseased spikelets and lower disease severity scores than those carrying *Fhb1* or *Fhb2* at a single gene level (Figure 3A). The DH lines with *Fhb1 + 5*, *Fhb2 + 5* and *Fhb1 + 2 + 5* showed better FHB resistance than those with single *Fhb5*. The DH lines carrying *Fhb1* exhibited better FHB resistance than those carrying *Fhb2* or *Fhb5* at a single gene level in the five single floret inoculation tests (*p* < 0.05). There was no difference between the lines carrying a single *Fhb5* gene and the three susceptible alleles. The DH lines carrying *Fhb1 + 2* or *Fhb1 + 2 + 5* had fewer diseased spikelets and lower disease severity scores than those carrying *Fhb1*, *Fhb2*, *Fhb5*, *Fhb1 + 5* and *Fhb2 + 5* in most of the tests, but there was no difference between the lines with *Fhb1 + 2* and *Fhb1 + 2 + 5*.

### 2.3. Performance of Main Agronomic Traits

**Figure 4:**
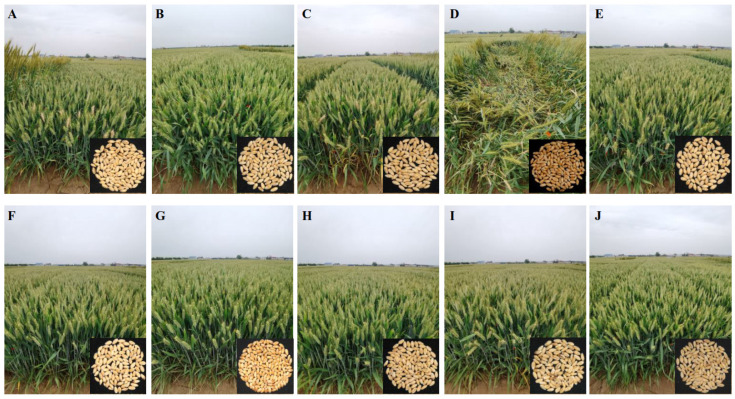
Field performances and kernels of the recipient and donor parents, controls and five promising double haploid (DH) lines. (**A**) Zhoumai 16; (**B**) Lunxuan 136; (**C**) Lunxuan 6; (**D**) Sumai 3; (**E**) Zhoumai 18; (**F**) DH 112 line; (**G**) DH 401 line; (**H**) DH 470 line; (**I**) DH 476 line; (**J**) DH 487 line.

**Figure 5:**
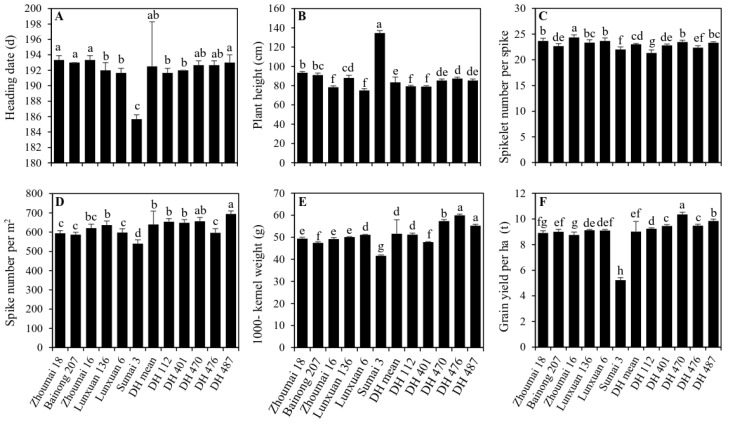
Comparison of heading date (**A**), plant height (**B**), spikelet number per spike (**C**), spike number per m^2^ (**D**), 1000-kernel weight (**E**) and grain yield per ha (**F**) among the controls, five selected DH lines and their parents. Multiple comparisons were performed using the least significant difference (LSD) test. Different letters above the standard deviation bars indicate significant difference among genotypes at *p* < 0.05.

Based on the evaluation of the FHB resistance in multiple tests, 49 FHB-resistant DH lines were evaluated for the main agronomic traits. Severe lodging occurred in the donor Sumai 3 at the anthesis date due to its taller plant height (Figure 4). The mean grain yield of the DH lines was not different from that of the recipient parents Lunxuan 136 and Lunxuan 6, but it was higher than that of the parent Zhoumai 16 and that of the donor parent Sumai 3 (Figure 5). Compared to the controls Zhoumai 18 and Bainong 207, the DH lines showed an earlier heading date, a lower plant height, a higher spike number and a higher thousand-kernel weight and grain yield, on average (*p* < 0.05). 

Five promising DH lines (DH 112, DH 401, DH 470, DH 476 and DH 487) were selected (Figure 4 and Figure 5). Compared to the controls Zhoumai 18 and Bainong 207, these lines had a lower plant height (Figure 5B), a higher kernel weight (Figure 5E) and a higher grain yield (Figure 5F) (*p* < 0.05). Most lines showed earlier or similar heading dates (Figure 5A) and similar and/or more spikelet numbers per spike (Figure 5C) and spike numbers (Figure 5D) when compared to the two controls. Different from the donor Sumai 3, these DH lines showed a higher yield potential, a shorter plant height and a better lodging resistance (*p* < 0.05). 

### 2.4. Molecular Marker and Genomic Composition Analyses of the Promising DH Lines 

Consistent with the recipient parents, the five DH lines (DH 112, DH 401, DH 470, DH 476 and DH 487) carried the semi-dwarfing gene *Rht-D1b* allele at the *Rht-D1* locus, and they shared the same recessive alleles (*vrn-A1*, *vrn-B1* and *vrn-D1*) for winter growth habit as the recurrent parents at the *Vrn-A1*, *Vrn-B1* and *Vrn-D1* loci, respectively (Supplementary Table S4). In contrast, Sumai 3 contained the *Rht-D1a* gene at the *Rht-D1* locus and the dominant allele *Vrn-D1a* at the *Vrn-D1* locus, showing a high plant height and spring growth habit. There were no allelic differences among the materials at the *Rht-B1* and *Vrn-B3* loci.

The genomic compositions of the five promising DH lines were analyzed with the Wheat 660 K SNP array (Supplementary Tables S5–S9). Approximately 80% of SNPs (with a range from 79.2% to 81.0%) for the DH lines were identical to the three recipient parents. The genetic contributions of Zhoumai 16, Lunxuan 136, Lunxuan 6 and Sumai 3 were 85.3%, 92,0%, 93,1% and 71.2% to line DH 112, 86.6%, 88.8%, 89.5%, and 73.2% to line DH 401, 86.5%, 92.3%, 92.8% and 71.7% to line DH 470, 85.9%, 88.9%, 92.2% and 71.0% to line DH 476 and 85.4%, 90.1%, 90.7% and 72.0% to line DH 487, respectively. Consistently, Lunxuan 6 provided higher genetic contributions than the other two recipient parents Zhoumai 16 and Lunxuan 136, but the latter two cultivars were higher than Sumai 3. In addition, 1.7%, 1.8%, 1.3%, 1.7% and 1.7% of the SNPs were unique to DH 112, DH 401, DH 470, DH 476 and DH 487, respectively.

## 3. Discussion

The mean value and standard deviation (SD) of each gene combination were calculated based on the DH lines within the gene combination. There was no entirely consistent resistance performance of all DH lines within a given gene combination. Such a difference in the resistance to FHB is attributed to the high SD values in most of the gene combinations (Figure 3). In the previous studies, we also found that the progenies carrying a single *Fhb1* gene also exhibited different FHB resistances [22,25,26]. 

The epidemics of FHB vary greatly between years in northern China, making it difficult to accurately evaluate the disease resistance in fields under natural infection, especially in a year with light disease incidence [20]. Molecular-marker-assisted selection can not only enhance the efficiency of selection but also shorten the breeding cycle. Previously, we tried to introgress *Fhb1* into winter wheat by means of the marker-assisted backcrossing strategy and found that *Fhb1* can only enhance the FHB resistance of winter wheats to a moderately susceptible level [22,25,26,27]. *Fhb2* and *Fhb5* were also effective in the improvement of FHB resistance in other spring or winter wheat genetic backgrounds [3,8,28]. *Fhb1*, *Fhb2*, *Fhb3*, *Fhb6* and *Fhb7* provide the Type II resistance, and *Fhb4* and *Fhb5* confer the Type I resistance. In this study, we showed that *Fhb1* or *Fhb2* were more efficient than *Fhb5* under single-floret infection tests, whereas *Fhb5* had a better FHB resistance than the genes *Fhb1* and *Fhb2* under the natural infection test. Jia et al. [8] also found that the lines carrying a single *Fhb1* gene had a significantly lower percentage of diseased spikelets than those carrying individual *Fhb4* or *Fhb5* after a single floret test; conversely, the single *Fhb4* or *Fhb5* showed a better FHB resistance than *Fhb1* under the natural infection test.

The stack of FHB resistance genes was more efficient and stable in increasing disease resistance compared to single gene carriers. The DH lines pyramiding of *Fhb1*, *Fhb2* and *Fhb5* showed better FHB resistance than those carrying single genes or other gene combinations (Figure 3). A possible reason is the additive effect of the three genes. Similar results were also reported by a number of the previous studies [3,8,24,28,29]. Even if the gene combination *Fhb1 + 2* showed a similar FHB resistance as *Fhb1 + 2 + 5* under the single-floret inoculation tests, its effect was lower than *Fhb1 + 5*, *Fhb2 + 5* and *Fhb1 + 2 + 5* under the natural infection test, demonstrating that the Type II resistance is not enough to protect against severe FHB epidemics, and the effective strategy is to combine the Type II resistance with the Type I resistance [30].

During the improvement of FHB resistance in winter wheat, it is difficult to obtain desirable traits using spring wheats developed in the MLWZ as donors [22,26]. For example, Sumai 3, a well-known FHB-resistant wheat cultivar, has a typical spring growth habit, a red grain color, a taller plant height and a low yield potential [26], and it is not popularly used by wheat breeders in northern China. Hence, the primary target for wheat breeders is to eliminate its undesirable traits and maintain its FHB resistance. It is necessary to enlarge the population size in order to select desirable plants. The dwarf-male-sterile (DMS) wheat can efficiently shorten breeding cycles by enlarging the population size to increase the possibility of ideal individuals based on the limited backcrossing due to the obviation of the labor-intensive emasculation step [27]. We have confirmed that *Fhb1* had no deleterious effect on agronomic performance in winter wheat [22]. However, Brar et al. [31] reported that the introduction of *Fhb5* led to a lower kernel weight and a slight increase in plant height. The three FHB resistance genes used in this study were introgressed into winter wheat cultivars, and most of the DH lines were significantly improved in the FHB resistance compared to the recipient parents and showed desirable performances in fields compared to the donor Sumai 3. Genomic composition analysis confirmed that more than 85% of SNPs (ranging from 85.3% to 93.1%) from the recipient parents were identical to their DH progenies, suggesting that it is possible to simultaneously improve FHB resistance and agronomic performance by the strategies of limited backcrossing or crossing in a large population size. This demonstrates that unfavorable linkage drags can be broken using a large population size combined with MAS, and *Fhb1*, *Fhb2* and *Fhb5* can effectively improve FHB resistance without a significant yield penalty. 

In conclusion, the findings from this study demonstrate that *Fhb1* is more efficient in improving FHB resistance than *Fhb2* and *Fhb5* at the single gene level under severe disease pressure provided by the single-floret inoculation, whereas *Fhb5* showed lower diseased spikelets than *Fhb1* and *Fhb2* under the natural inoculation test. Pyramiding *Fhb1*, *Fhb2* and *Fhb5* showed a better FHB resistance. Five promising DH lines with moderate resistance to FHB and promising agronomic traits were selected, one of which, DH 112, has been involved in the Winter Wheat Regional Trials in Henan province.

## 4. Materials and Methods

### 4.1. Plant Materials

A DH population was constructed based on the MAS combined with the DMS wheat (Supplementary Figure S1). The DMS-Zhoumai 16/Sumai 3//Zhoumai 16 BC_1_F_1_ population was developed in our previous study [26]. The BC_1_F_1_ DMS plants were continually crossed with the Zhoumai 16-derived cultivars Lunxuan 136 (pedigree: Zhoumai 16/Zhengmai 9023//Zhoumai 16) and Lunxuan 6 (pedigree: Aikang 58/Lunxun 136) to develop a four-way F_1_ hybrid (Zhoumai 16/Sumai 3//Zhoumai 16/3/Lunxuan 136/4/Lunxuan 6). The normal male fertile plants from the Zhoumai 16/Sumai 3//Zhoumai 16/3/Lunxuan 136/4/Lunxuan 6 population were selected to generate the DH lines via the wheat by the maize (*Zea mayz* L.) cross technique. The wheat cultivars Sumai 3, Yangmai 158, Huaimai 20 and Zhoumai 16 were used as the highly resistant, moderately resistant, moderately susceptible and highly susceptible controls, respectively. The recipient parents Zhoumai 16, Lunxuan 136 and Lunxuan 6, the donor parent Sumai 3 and the control cultivars Zhoumai 18 (a control of the National Wheat Regional Trials in the southern Yellow and Huai River Valleys Winter Wheat Zone) and Bainong 207 (a control of the Henan Wheat Regional Trails) were used as the control cultivars during the evaluation of agronomic traits. 

### 4.2. Molecular Marker Detection of Genes for Resistance to FHB

The resistance and susceptibility alleles of *Fhb1* were differentiated by observing the PCR products with its gene-specific primers for the marker (GSM) *TaHRC-GSM* [32]. Four gene-linked single sequence repeat (SSR) markers (*Xwmc397* and *Xwmc398*) were used to determine *Fhb2* [33]. The markers *Xgwm304*, *Xhbg394* and *Wmc705* were used to detect *Fhb5* [2,34]. The primer information of all the markers is listed in Supplementary Table S10. The primers specific for genes conferring plant height and vernalization were used to differentiate the alleles in recurrent parents, donors and promising DH lines according to the methods described by Zhang et al. [35]. Polymerase chain reaction (PCR) was performed with an initial denaturation at 94 °C for 5 min, 35 cycles of 94 °C for 30 s and annealing at 60–64 °C for 30 s and 72 °C for 30 s–2 min, with a final extension at 72 °C for 10 min. The *TaHRC-GSM* PCR products were separated by electrophoresis in 2% agarose gels for 30 min and visualized by staining with GeneFinder (Bio-V, Xiamen, China). The other PCR products were separated in 8% non-denaturing polyacrylamide gels and visualized by silver staining.

### 4.3. Assessments of FHB Resistance

A single-floret inoculation method was performed to determine the disease resistance at the Xinxiang Experimental Station (35°31′ N and 113°85′ E) of the Chinese Academy of Agricultural Sciences (CAAS) in Henan province in 2021 and 2022 (2021HN and 2022HN, respectively), the Beijing Dongpuchang Experimental Station (39°95′ N and 116°30′ E) of CAAS in Beijing in 2021 (2021BJ) and the greenhouse of the Institute of Crop Sciences, CAAS in Beijing in 2021 and 2022 (2021GH and 2022GH, respectively). All the DH lines and controls were arranged in a randomized complete block design with two replicates. Each entry was sown in a 2 m row with 40 seeds at 2021HN, 2021BJ and 2021HN tests. Ten seedlings of each entry were sown in each pot (25 cm in diameter) in the greenhouse at 2021GH and 2022 GH tests. The macroconidia inoculum was prepared according to the method described by Bai and Shaner [36]. Ten spikes per entry with the same flowering time were injected with 10 μL (~1000 spores) of *F. graminearum* conidiospore suspension into the central spikelets of spikes at the early stage of anthesis. The inoculated spikes were sprayed with water, covered with plastic bags for 48 h and overhead mist irrigated to maintain moisture until 15 d post-inoculation (dpi). After 21 dpi, the total number of diseased spikelets was counted to calculate the disease severity [37,38].

The natural disease nursery was established at the Experimental Farm of Nanping Academy of Agricultural Science (27°33′ N, 118°12′ E) in Fujian province to perform the field assessment of FHB resistance during the 2019/2020 cropping season (2020FJ). All entries were arranged in a randomized complete block design with two replicates. Each entry was sown in a 1 m row with 20 seeds per row. The number of infected spikelets and the total number of spikelets on 10 spikes from each entry were counted 25 d after anthesis [22]. 

### 4.4. Observation of Agronomic Traits 

During the 2020/2021 wheat cropping season, a field trial was performed to investigate the agronomic traits of selected lines at the Xinxiang Experimental Station of CAAS in Henan using a randomized complete block design with three replicates. Each plot consisted of six rows that were 6 m in length, with 0.2 m between rows. The heading date (d), plant height (cm), spikelet number per spike, spike number m^−2^, 1000-kernel weight (g) and grain yield (t ha^−1^) were investigated according to the methods reported by Meng et al. [39]. 

### 4.5. Genomic Composition Analysis 

The recurrent parents, donors and five selected DH lines were genotyped using the Affymetrix Wheat 660 K SNP array developed by the Institute of Crop Sciences, CAAS at CapitalBio Technology Co., Ltd., Beijing, China. Raw data were processed by the Axiom Analysis Suite software (version 3.1.51) (Thermo Fisher Scientific-CN Co., Ltd., Shanghai, China). Sequences of SNPs were blasted against the Chinese Spring reference genome sequences (IWGSC RefSeq v1.0) to determine their chromosomal and physical locations. High-quality SNPs were obtained to analyze the genomic compositions of DH lines by removing markers without chromosomal locations and missing genotype information.

### 4.6. Statistical Analysis

The data analysis tool in Microsoft Excel 2019 was used to analyze the correlations and determine the descriptive statistical analysis including mean values and ranges. ANOVA was performed in IBM SPSS Statistics 22 (International Business Machines Corporation, Armonk, NY, USA) using a mixed linear model with the DH lines and tests as the fixed effects and a block within each test as the random effect according to the method described by Dixon et al. [40]. The least significant difference (LSD) at *p* < 0.05 was used to perform the multiple comparisons of FHB resistance and agronomic traits using IBM SPSS Statistics 22 (International Business Machines Corporation, Armonk, NY, USA).

## Data Availability

All data generated or analyzed during this study are included in this published article.

## Supplementary Materials

The following supporting information can be downloaded at: https://www.mdpi.com/article/10.3390/ijms232315047/s1.

## References

[1] Bai G.H., Shaner G. (2004). Management and resistance in wheat and barley to *Fusarium* head blight. Annu Rev. Phytopathol..

[2] Buerstmayr M., Sterner B., Wagner C., Schwarz P., Brugger K., Barabaschi D., Volante A., Giampiero V., Cattivelli L., Buerstmayr H. (2018). High-resolution mapping of the pericentromeric region on wheat chromosome arm 5AS harboring the *Fusarium* head blight resistance QTL *Qfhs.ifa-5A*. Plant Biotech. J..

[3] Ma Z.Q., Xie Q., Li G.Q., Jia H.Y., Zhou J.Y., Kong Z.X., Li N., Yuan Y. (2020). Germplasms, genetics and genomics for better control of disastrous wheat *Fusarium* head blight. Theor. Appl. Genet..

[4] He X., Dreisigacker S., Singh R.P., Singh P.K. (2019). Genetics for low correlation between *Fusarium* head blight disease and deoxynivalenol (DON) content in a bread wheat mapping population. Theor. Appl. Genet..

[5] Jin F., Zhang D.D., Bockus W., Baenziger P.S., Carver B., Bai G.H. (2013). *Fusarium* head blight resistance in U.S. winter wheat cultivars and elite breeding lines. Crop Sci..

[6] Chen Y., Kistler H.C., Ma Z.H. (2019). *Fusarium graminearum* trichothecene mycotoxins: Biosynthesis, regulation and management. Annu. Rev. Phytopathol..

[7] Li G.Q., Zhang J.Y., Jia H.Y., Guo Z.X., Fan M., Luo Y.J., Zhao P.T., Xue S.L., Li N., Yuan Y. (2019). Mutation of a histidine-rice calcium-binding-protein gene in wheat confers resistance to *Fusarium* head blight. Nat. Genet..

[8] Jia H.Y., Zhou J.Y., Xue S.L., Li G.Q., Yan H.S., Ran C.F., Zhang Y.D., Shi J.X., Jia L., Wang X. (2018). A journey to understand wheat *Fusarium* head blight resistance in Chinese wheat landrace Wangshuibai. Crop J..

[9] Qi L.L., Pumphrey M.Q., Friebe B., Chen P.D., Gill B.S. (2008). Molecular cytogenetic characterizaiton of alien introgression with gene *Fhb3* for resistance to *Fusarium* head blight disease of wheat. Theor. Appl. Genet..

[10] Cainong J.C., Bockus W.W., Feng Y., Chen P., Qi L., Sehgal S.K., Danilova T.V., Koo D.H., Friebe B., Gill B.S. (2015). Chromosome engineering, mapping, and transferring of resistance to *Fusarium* head blight disease from *Elymus tsukushiensis* into wheat. Theor. Appl. Genet..

[11] Guo J., Zhang X.L., Hou Y.L., Cai J.J., Shen X.R., Zhou T.T., Xu H.H., Ohm H.W., Wang H.W., Li A.F. (2015). High-density mapping of the major FHB resistance gene *Fhb7* derived from *Thinopyrum ponticum* and its pyramiding with *Fhb1* by marker-assisted selection. Theor. Appl. Genet..

[12] Bai G.H., Su Z.Q., Cai J. (2018). Wheat resistance to *Fusarium* head blight. Can. J. Plant Pathol..

[13] Rawat N., Pumphrey M.O., Liu S., Zhang X., Tiwari V.K., Ando K., Trick H.N., Bockus W.W., Akhunov E., Anderson J.A. (2016). Wheat *Fhb1* encodes a chimeric lectin with agglutinin domains and a pore-forming toxin-like domain conferring resistance to *Fusarium* head blight. Nat. Genet..

[14] Su Z.Q., Bernardo A., Tian B., Chen H., Wang S., Ma H.X., Cai S.B., Liu D.T., Zhang D.D., Li T. (2019). A deletion mutation in *TaHRC* confers *Fhb1* resistance to *Fusarium* head blight in wheat. Nat. Genet..

[15] Paudel B., Zhuang Y., Galla A., Dahal S., Qiu Y., Ma A., Raihan T., Yen Y. (2020). *WFhb1-1* plays an important role in resistance against *Fusarium* head blight in wheat. Sci. Rep..

[16] Wang H.W., Sun S.L., Ge W.Y., Zhao L.F., Hou B.Q., Wang K., Lyu Z.F., Chen L.Y., Xu S.S., Guo J. (2020). Horizontal gene transfer of *Fhb7* from fungus underlies *Fusarium* head blight resistance in wheat. Science.

[17] Dou L., Li Z., Shen Q., Shi H., Li H., Wang W., Zou C., Shang H., Li H., Xiao G. (2021). Genome-wide characterization of the *WAK* gene family and expression analysis under plant hormone treatment in cotton. BMC Genom..

[18] Giancaspro A., Lionetti V., Giove S.L., Zito D., Fabri E., Reem N., Zabotina O.A., Angelis E.D., Monaci L., Bellincampi D. (2018). Cell wall features transferred from common into durum wheat to improve *Fusarium* head blight resistance. Plant Sci..

[19] Gadaleta A., Colasuonno P., Giove S.L., Blanco A., Giancaspro A. (2019). Map-based cloning of *QFhb.mgb-2A* identifies a *WAK2* gene responsible for *Fusarium* head blight resistance inwheat. Sci. Rep..

[20] Zhu Z.W., Hao Y.F., Mergoum M., Bai G.H., Humphreys G., Cloutier S., Xia X.C., He Z.H. (2019). Breeding wheat for resistance to *Fusarium* head blight in the Global North: China, USA, and Canada. Crop J..

[21] Clark A.J., Sarti-Dvorjak D., Brown-Guedira G., Dong Y., Baik B.K., van Sanford D.A. (2016). Identifying rare FHB-resistant segregants in intransigent backcross and F_2_ winter wheat populations. Front. Microbiol..

[22] Li T., Zhang H.J., Huang Y.W., Su Z.Q., Deng Y., Liu H.W., Mai C.Y., Yu G.J., Li H.L., Yu L.Q. (2019). Effects of the *Fhb1* gene on *Fusarium* head blight resistance and agronomic traits of winter wheat. Crop J..

[23] Buerstmayr M., Steiner B., Buerstmayr H. (2020). Breeding for *Fusarium* head blight resistance in wheat—Progress and challenges. Plant Breed..

[24] Zhang Y.D., Yang Z.B., Ma H.C., Huang L.Y., Ding F., Du Y.Y., Jia H.Y., Li G.Q., Kong Z.X., Ran C.F. (2021). Pyramiding of *Fusarium* head blight resistance quantitative trait loci, *Fhb1*, *Fhb4*, and *Fhb5*, in modern Chinese wheat cultivars. Front. Plant Sci..

[25] Dai X.R., Huang Y.W., Li T., Deng Y., Su Y., Mai C.Y., Yu L.Q., Li H.L., Liu H.W., Yang L. (2021). Improvement of resistance to *Fusarium* head blight by *Fhb1* molecular marker-assisted backcrossing in the Huang-Huang River Valley Winter Wheat Region. J. Triticeae Crops.

[26] Zhang H.J., Su Z.Q., Bai G.H., Zhang X., Ma H.Q., Li T., Deng Y., Mai C.Y., Yu L.Q., Liu H.W. (2018). Improvement of resistance of wheat cultivars to *Fusarium* head blight in the Yellow-Huai River Valleys Winter Wheat Zone with functional marker selection of *Fhb1* gene. Acta Agron. Sin..

[27] Zhou Y., Zhang H.J., Wang C.Y., Li H.J., Mai C.Y., Yang L., Liu H.W., Yu L.Q., Yu G.J., Liu B.H. (2022). Dwarf-male-sterile wheat and its application in breeding program for *Fusarium* head blight resistance in Southern Yellow and Huai River Valleys Winter Wheat Region. Acta Agron. Sin..

[28] Brar G.S., Brûlé-Babel A.L., Ruan Y.F., Henriquez M.A., Pozniak C.J., Kutcher H.R., Hucl P.J. (2019). Genetic factors affecting *Fusarium* head blight resistance improvement from introgression of exotic Sumai 3 alleles (including *Fhb1*, *Fhb2*, and *Fhb5*) in hard red spring wheat. BMC Plant Biol..

[29] Hu W.J., Fu L.P., Cao D.R., Li D.S., Liao S., Lu C.B. Marker-assisted selection to pyramid *Fusarium* head blight resistance loci *Fhb1* and *Fhb2* in a high-quality soft wheat cultivar Yangmai 15. J. Integr. Agr..

[30] Rudd J.C., Horsley R.D., McKendry A.L., Elias E.M. (2001). Host plant resistance genes for *Fusarium* head blight: Sources, mechanisms, and utility in conventional breeding systems. Crop Sci..

[31] Brar G.S., Pozniak C.J., Kutcher H.R., Hucl P.J. (2019). Evaluation of *Fusarium* head blight resistance genes *Fhb1*, *Fhb2*, and *Fhb5* introgressed into elite Canadian hard red spring wheats: Effect on agronomic and end-use quality traits and implications for breeding. Mol. Breed..

[32] Su Z.Q., Jin S.J., Zhang D.D., Bai G.H. (2018). Development and validation of diagnostic markers for *Fhb1* region, a major QTL for *Fusarium* head blight resistance in wheat. Theor. Appl. Genet..

[33] Cuthbert P.A., Somers D.J., Brulé-Babel A. (2007). Mapping of *Fhb2* on chromosome 6BS: A gene controlling *Fusarium* head blight field resistance in bread wheat (*Triticum aestivum* L.). Theor. Appl. Genet..

[34] Xue S.L., Xu F., Tang M.Z., Zhou Y., Li G.Q., An X., Lin F., Xu H.B., Jia H.Y., Zhang L.X. (2011). Precise mapping *Fhb5*, a major QTL conditionaing resistance to *Fusarium* infection in bread wheat (*Triticum aestivum* L.). Theor. Appl. Genet..

[35] Zhang H.J., Xue X.H., Guo J., Huang Y.W., Dai X.R., Li T., Hu J.H., Qu Y.F., Yu L.Q., Mai C.Y. (2022). Association of the recessive allele *vrn-D1* with winter frost tolerance in bread wheat. Front. Plant Sci..

[36] Bai G.H., Shaner G. (1996). Variation in *Fusarium graminearum* and cultivar resistance to wheat scab. Plant Dis..

[37] Bai G.H., Kolb F.L., Shaner G., Domier L.L. (1999). Amplified fragment length polymorphism markers linked to a major quantitative trait locus controlling scab resistance in wheat. Phytopathology.

[38] Zhang X., Jiang P., Ye R.Y., Wu L., Zhang Y., Ma H.X. (2017). Evaluation and source tracing of resistance of *Fusarium* head blight in wheat variety Ningmai 9 and its derivatives. Mol. Plant Breed..

[39] Meng L.Z., Liu H.W., Yang L., Mai C.Y., Yu L.Q., Li H.J., Zhang H.J., Zhou Y. (2016). Effects of the *Vrn-D1b* allele associated with facultative growth habit on agronomic traits in common wheat. Euphytica.

[40] Dixon P.M., Moore K.J., Santen E.V., Glaz B., Yeater K.M. (2018). The analysis of combined experiments. Applied Statistics in Agricultura, Biolgica, and Environmental Siences.

