# Supplementary material for: Effects of Fhb1, Fhb2 and Fhb5 on Fusarium Head Blight Resistance and the Development of Promising Lines in Winter Wheat
Source: Int J Mol Sci. 2022 Nov 30;23(23):15047. doi: 10.3390/ijms232315047 (PMC9739584; doi:10.3390/ijms232315047)
Supplement: Supplementary file 1 [file ijms-23-15047-s001.zip › Table S1.pdf]

Supplementary Table S1 Number of double haploid (DH) lines used for the evaluation of *Fusarium* head blight (FHB) resistance at six tests

Genotype	Number of DH lines					
	2020FJ	2021HN	2021BJ	2021GH	2022HN	2022GH
None	10	30	30	30	30	30
Fhb1	10	30	30	30	30	30
Fhb2	10	30	30	30	30	30
Fhb5	10	30	30	30	30	30
Fhb1 + 2	10	30	30	30	30	30
Fhb1 + 5	10	26	26	26	26	26
Fhb2 + 5	10	30	30	30	30	30
Fhb1 + 2 + 5	6	11	11	11	11	11
