# Supplementary material for: Effects of Fhb1, Fhb2 and Fhb5 on Fusarium Head Blight Resistance and the Development of Promising Lines in Winter Wheat
Source: Int J Mol Sci. 2022 Nov 30;23(23):15047. doi: 10.3390/ijms232315047 (PMC9739584; doi:10.3390/ijms232315047)
Supplement: Supplementary file 1 [file ijms-23-15047-s001.zip › Table S10.pdf]

Supplementary Table S10 The primer information of all markers used in identification of genes *Fhb1*, *Fhb2* and *Fhb5*

Gene	Locus	Chromosome	Primer sequence (5′-3′)	Fragment size (bp)	Tm (°C)	Reference
Fhb1	*TaHRC*	3B	F: ATTCCTACTAGCCGCCTGGT	1400	64	Su et al. (2018)
			R: ACTGGGGCAAGCAAACATTG			
Fhb2	*Wmc397*	6B	F: AGTCGTGCACCTCCATTTTG	155	61	Cuthbert et al. (2007)
			R: CATTGGACATCGGAGACCTG			
	Wmc398	6B	F: GGAGATTGACCGAGTGGAT	160	61	Cuthbert et al. (2007)
			R: CGTGAGAGCGGTTCTTTG			
Fhb5	*Gwm304*	5A	F: AGGAAACAGAAATATCGCGG	220	61	Xue et al. (2011)
			R: AGGACTGTGGGGAATGAATG			
	Xhbg394	5A	F: CCACAACGAAGAACTGCTTACA	270	60	Xue et al. (2011)
			R: AGCTAGATGCTTCCTAGAGTGGAG			
	Wmc705	5A	F: GGTTGGGCTCCTGTCTGTGAA	160	67	Somers et al. (2005)
			R: TCTTGCACCTTCCCATGCTCT			
