# Supplementary material for: Effects of Fhb1, Fhb2 and Fhb5 on Fusarium Head Blight Resistance and the Development of Promising Lines in Winter Wheat
Source: Int J Mol Sci. 2022 Nov 30;23(23):15047. doi: 10.3390/ijms232315047 (PMC9739584; doi:10.3390/ijms232315047)
Supplement: Supplementary file 1 [file ijms-23-15047-s001.zip › Table S2.pdf]

Supplementary Table S2 Analysis of variance for number of diseased spikelets and disease severity in the DH lines at multiple tests.

Source	Degree of freedom	Number of diseased spikelets			Disease severity		
		Mean square	*F*-value	*P*-value	Mean square	*F*-value	*P*-value
Test (T)[a]	4	5652.1	973.5	<0.001	139971.8	1059.4	<0.001
Block (Test)	5	96.2			2316.6		
Genotype (G)	216	125.3	21.6	<0.001	2849.4	21.6	<0.001
T × E	845	19.7	3.4	<0.001	460.4	3.5	<0.001
Error	1069						

[a] Data of 2020FJ unincluded.
