# Supplementary material for: Effects of Fhb1, Fhb2 and Fhb5 on Fusarium Head Blight Resistance and the Development of Promising Lines in Winter Wheat
Source: Int J Mol Sci. 2022 Nov 30;23(23):15047. doi: 10.3390/ijms232315047 (PMC9739584; doi:10.3390/ijms232315047)
Supplement: Supplementary file 1 [file ijms-23-15047-s001.zip › Table S3.pdf]

Supplementary Table S3 Correlation coefficients of number of diseased spikelets (bottom left) or disease severity (top right) in different tests.

Environment [a]	2021HN	2021BJ	2021GH	2022HN	2022GH
2021HN		0.84**	0.60**	0.64**	0.49**
2021BJ	0.84**		0.61**	0.59**	0.46**
2021GH	0.64**	0.64**		0.46**	0.55**
2022HN	0.64**	0.59**	0.45**		0.30**
2022GH	0.50**	0.46**	0.56**	0.29**	

[a] Data of 2020FJ unincluded. **: Significant at $P < 0.01$.
