# Supplementary material for: Effects of Fhb1, Fhb2 and Fhb5 on Fusarium Head Blight Resistance and the Development of Promising Lines in Winter Wheat
Source: Int J Mol Sci. 2022 Nov 30;23(23):15047. doi: 10.3390/ijms232315047 (PMC9739584; doi:10.3390/ijms232315047)
Supplement: Supplementary file 1 [file ijms-23-15047-s001.zip › Table S4.pdf]

Supplementary Table S4 Allelic variation at the loci associated with plant height and vernalization identified in the best five double haploid (DH) lines and their parents

Parent/Line	*Rht-B1*	*Rht-D1*	*Vrn-A1*	*Vrn-B1*	*Vrn-D1*	*Vrn-B3*
Parents						
Zhoumai 16	*Rht-B1a*	*Rht-D1b*	*vrn-A1*	*vrn-B1*	*vrn-D1*	*vrn-B3*
Lunxuan 136	*Rht-B1a*	*Rht-D1b*	*vrn-A1*	*vrn-B1*	*vrn-D1*	*vrn-B3*
Lunxuan 6	*Rht-B1a*	*Rht-D1b*	*vrn-A1*	*vrn-B1*	*vrn-D1*	*vrn-B3*
Sumai 3	*Rht-B1a*	*Rht-D1a*	*vrn-A1*	*vrn-B1*	*Vrn-D1a*	*vrn-B3*
Lines						
DH 112	*Rht-B1a*	*Rht-D1b*	*vrn-A1*	*vrn-B1*	*vrn-D1*	*vrn-B3*
DH 401	*Rht-B1a*	*Rht-D1b*	*vrn-A1*	*vrn-B1*	*vrn-D1*	*vrn-B3*
DH 470	*Rht-B1a*	*Rht-D1b*	*vrn-A1*	*vrn-B1*	*vrn-D1*	*vrn-B3*
DH 476	*Rht-B1a*	*Rht-D1b*	*vrn-A1*	*vrn-B1*	*vrn-D1*	*vrn-B3*
DH 487	*Rht-B1a*	*Rht-D1b*	*vrn-A1*	*vrn-B1*	*vrn-D1*	*vrn-B3*
