# Supplementary figures and images for: Effects of Fhb1, Fhb2 and Fhb5 on Fusarium Head Blight Resistance and the Development of Promising Lines in Winter Wheat
Source: Int J Mol Sci. 2022 Nov 30;23(23):15047. doi: 10.3390/ijms232315047 (PMC9739584; doi:10.3390/ijms232315047)

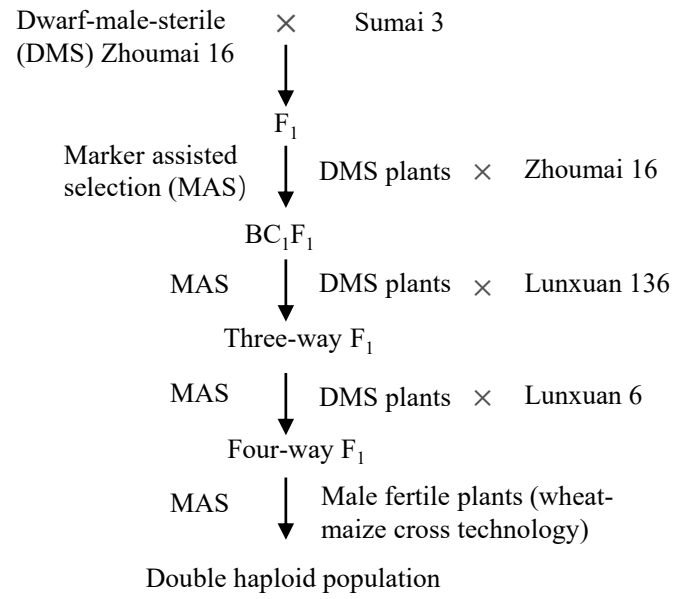

Supplement: Supplementary file 1 [file ijms-23-15047-s001.zip › Figure S1.pdf]
